# Left Atrial Strain Value Versus Tissue Doppler Echocardiography and the Left Atrium Volume Index in the Evaluation of Left Ventricular Diastolic Function in Patients with Chronic Kidney Disease

**DOI:** 10.3390/clinpract15020036

**Published:** 2025-02-13

**Authors:** Eman Elsheikh, Zainab Amjad, Samah I Abohamr, Muthana Al Sahlawi, Ibtsam Khairat

**Affiliations:** 1 Cardiovascular Department, Faculty of Medicine, Tanta University, Tanta 31527, Egypt; s.abohamar@ksmc.med.sa (S.I.A.); or basma_cardiotanta@hotmail.com (I.K.); 2 Internal Medicine Department, College of Medicine, King Faisal University, Alahsa 31982, Saudi Arabia; zaali@kfu.edu.sa; 3 Heart Health Centre, King Saud Medical City, Riyadh 12372, Saudi Arabia

**Keywords:** left atrial strain, tissue doppler, echocardiography, chronic kidney disease, left atrium volume index, diastolic function

## Abstract

**Background:** In heart failure with preserved ejection fraction (HFpEF) and decreased ejection fraction (HFrEF), LA strain, an indicator of the filling and discharge of the left atrium (LA), was recently identified as a sign of diastolic dysfunction. Our objective was to examine the significance of left atrial (LA) strain relative to tissue Doppler echocardiography and the left atrial volume index (LAVI) in evaluating left ventricular (LV) diastolic performance in individuals with chronic kidney disease (CKD). **Methods**: A prospective cross-sectional study was conducted on 220 outpatients with CKD who fulfilled the inclusion criteria and were referred to the cardiology clinic at Tanta University for routine echocardiographic assessment during a period of 6 months (April to September 2024). Based on their estimated glomerular filtration rate (eGFR), patients were divided into five groups: GFR ranges from 90 to 120 mL/min/1.73 m^2^ in Group 1, 60 to 90 mL/min/1.73 m^2^ in Group 2, 30 to 60 mL/min/1.73 m^2^ in Group 3, 15 to 29 mL/min/1.73 m^2^ in Group 4, and less than 15 mL/min/1.73 m^2^ in Group 5. All participants were evaluated using echocardiographic measurements, such as the E/e ratio, left ventricular systolic and diastolic volumes, left atrial strain, left atrial volume index (LAVI), and ejection fraction (EF). **Results**: LA strain conduit and reservoir strain can significantly diagnose LV diastolic function in CKD patients (*p* < 0.001; AUC = 0.819 and 0.869, respectively) using cutoffs of ≤36 and ≥23, with 86% and 95.5% sensitivity, 65% and 60% specificity, 96.1% and 96% PPV, and 31.7% and 57.1%, NPV respectively. An AUC = 0.926 was observed with LAVI among grade 1 vs. grade 0 (0.9 is considered excellent in diagnosing patients with and without the disease). Other markers had AUC values of 0.5–0.6 among the grades of the diastolic function, suggesting no discrimination in diagnosing the disease. **Conclusions:** LA conduit strain and reservoir strain are independent markers that represent a superior and more sensitive approach than LAVI and tissue Doppler echocardiography for evaluating LV diastolic dysfunction in patients with CKD, even in the early stages.

## 1. Introduction

Chronic kidney disease (CKD) is a major health condition that affected 697.5 million people globally and caused 1.2 million deaths annually in 2017. CKD individuals are universally acknowledged to possess an elevated risk of cardiovascular disease (CVD) [1].

In a certain regard, CKD is a cardiovascular risk factor that is equivalent to all-cause mortality and may be considered a separate condition from coronary artery disease (CAD). However, chronic renal illness may progress more quickly to end-stage kidney disease (ESKD) as a result of cardiovascular problems [2].

Additionally, studies show CVD takes more lives among people with CKD than other causes of death especially when the estimated glomerular filtration rate (eGFR) is at or below 60 mL/min/1.73 m^2^. People with CKD face a doubled to tripled risk of death because ischemic heart disease and heart failure (HF) [3] lead to 45% more cardiovascular deaths and 23% more deaths from all causes when they develop atrial fibrillation [4].

Evidence of left ventricular diastolic dysfunction (LVDD) is necessary to diagnose HF with preserved ejection fraction (HFpEF) in the general population, including its preclinical stage. LVDD serves as an autonomous prognostic indicator of all-cause mortality [5]. Therefore, it is becoming increasingly crucial to accurately assess LVDD in the routine clinical practice context. The primary physiologic consequence of LVDD is increased LV filling pressures. Echocardiography (Echo) is a frequently used noninvasive alternative to invasive procedures, which are regarded the “gold standard” for measuring left ventricular (LV) filling pressures and diastolic function [5].

The 2016 ASE/SCAI guidelines make it easier to evaluate left ventricular diastolic performance by combining four factors into one approach. The presence of substantial tricuspid valve abnormalities, pulmonary arterial hypertension, and low filling pressures in the right ventricle and right atrium will negatively impact the accuracy of the evaluation. Transthoracic echocardiography is an important tool for accurately assessing left ventricular diastolic function [5].

Speckle tracking is a technique that properly represents the intrinsic deformation of the left atrium (LA). It is less impacted by load fluctuations because of its relatively independent load circumstances and geometric structure. It is recommended that the LA strain be employed in the LVDD diagnosis [6].

LA strain is a measure of the filling and discharge of the LA, and it has been shown that HF with HFpEF and decreased ejection fraction (HFrEF) represent a measure of diastolic dysfunction. Conduit strain, reservoir strain, and booster (pump) strain are the three LA function phases that could be quantified using speckle-tracking analysis during Echo [7].

For those who are not diagnosed with CVD, lower LA strain values (which indicate poorer LA function) are contrary outcomes as indicated in [8] and observed in HFpEF patients [9]. Even though diastolic dysfunction and HFpEF are common in ESRD individuals, LA strain may be a useful prognostic indicator for this group. Research on LA strain in this group is, nonetheless, scarce.

There has been an evidence dearth regarding the distinctions in LA and LV strain and size changes across CKD stages, as well as the echocardiographic parameters that could be used to predict GFR decline. Hence, we specifically aimed to address the role of LA strain value versus tissue Doppler Echo and the LA volume index in the LV diastolic function evaluation in CKD patients.

## 2. Methodology

It was a cross-sectional study done on 220 CKD patients seen at the Cardiology Department of Tanta University for routine evaluation during a period of 6 months (April to September 2024). The anonymity of the study participants was maintained. CKD patients with EF > 50% were included. Patients having a history of myocardial dysfunction, dilated cardiomyopathy, congenital heart disease, ischemic heart disease, valvular heart disease, chronic obstructive pulmonary disease, and an ejection fraction below 50% were not allowed to participate in the research. Based on their eGFR, which was determined using the Cockcroft–Gault equation for creatinine clearance, the research participants were split up into five groups. A GFR of 90–120 mL/min/1.73 m^2^ is seen in Group 1, 60–90 mL/min/1.73 m^2^ in Group 2, 30–60 mL/min/1.73 m^2^ in Group 3, 15–29 mL/min/1.73 m^2^ in Group 4, and less than 15 mL/min/1.73 m^2^ in Group 5 [10]. The research protocol was authorized by Tanta University’s Ethics Committee in Egypt (reference number: 36264PR597/3/24, 09-03-2024).

## 3. Echocardiography

**Figure 1:**
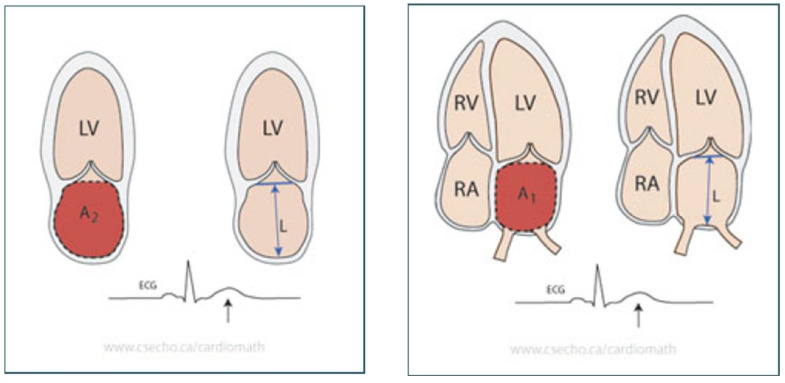
Assessment of LAV using the biplane area-length method [11]. A1 is the maximum area in the apical four-chamber view, where the left atrial area is planimetered. A2 is the planimetered left atrial area in the apical two-chamber view. L (length) is the distance in centimeters between the line between the hinge points of the mitral valve and the posterior wall. LAVI is the index of left atrial volume to body surface area.

All patients were scheduled for Echo using a Vivid E9 ultrasound system (GE Vingmed, Horton, Norway). All acquisitions were done with a broad-band M5S transducer (2.5–5 MHz) with the patient in the left lateral position utilizing apical four chambers, apical views, and parasternal short axis and long axis views following the American Society of Echo (ASE) recommendations [11]. The ECG was connected to establish the timing of cardiac cycle events. The ASE’s recommendations were followed in the execution of two-dimensional Echo. The two-dimensional M-mode method was employed to measure EF, and the *LA* was assessed by examining its dimensions and volumes [11]. The parasternal long axis image’s anteroposterior diameter is assessed using M-mode. The biplane area-length method was used to calculate the volumes of the *LA*. The maximal left atrial size was measured at the end of the T wave on the ECG, at the end of ventricular systole, or just before to the mitral valve opening. The pulmonary veins and the confluences of the left atrial appendage were excised during the planimetry process. The distance between the superior aspect of the left atrial (*LA*) and the midway of the mitral annulus plane, perpendicular to the plane, is known as the *LA* long-axis length, or *L*. The length is evaluated from the four-chamber and two-chamber perspectives using the area-length formula. As shown in Figure 1, this approach uses the shorter of the two length measures [12].

$$LA\;Volume=\frac{8}{3\pi }\times \frac{A1\times A2}{L}=0.85\times \frac{A1\times A2}{L}$$

$$LAVI=\frac{LA\;Volume}{Body\;surface\;area}$$

Using pulsed-wave Doppler, the transmitral blood flow was recorded in the apical four-chamber view to evaluate diastolic function. We assessed the E/A ratio, the E wave deceleration time (DT), the late diastolic blood flow rate (mitral late filling peak velocity, A), and the E wave rate. The maximum early flow (e′) in the lateral and septal annulus was evaluated using tissue Doppler, and the E/e′ ratio was calculated.

## 4. LA Longitudinal Strain Analysis

**Figure 2:**
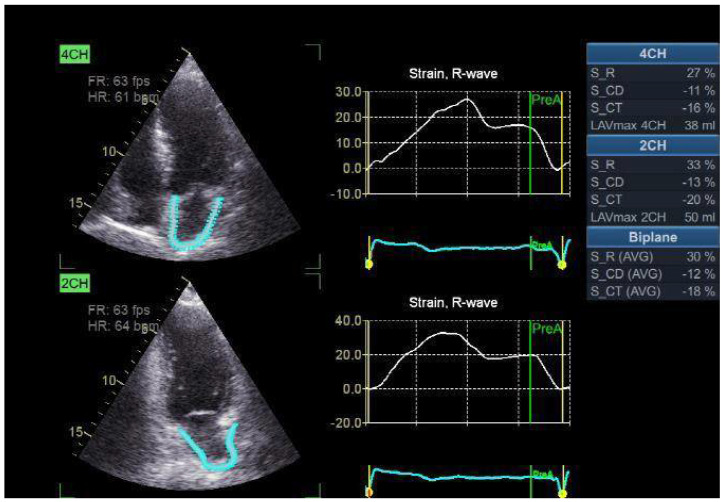
LA two-dimensional strain. LASr is assessed as the difference between onset of filling and end-diastole; LAScd is assessed as the difference between atrial contraction and filling onset; LASct is assessed as the difference between atrial filling onset and end-diastole.

The two-dimensional strain analysis tool on the Echo PAC workstation (GE Healthcare) was used to measure LA strain and strain rate. The endocardial border outlines the whole myocardial area of interest (ROI) of the left atrium, much as it does for the left ventricle. With a default width of 3 mm, it is advised to use an adjustable ROI. The item’s size and location may be altered by the operator to meet the left atrial wall’s thickness without touching the pericardium. After dividing the atrial endocardium into six segments, the computer automatically rejected and removed parts that were not presented properly from the research. The software created the global longitudinal strain and strain rate profiles for each apical view. In order to accomplish appropriate tracking, the operator has the option of either retrying the imaging process or modifying the software settings, such as the smoothing algorithms and the ROI width. The electrocardiograph was recorded during three consecutive heartbeats, and the apical four-chamber and two-chamber images were acquired. The absolute strain value in the three LA phases—conduit strain in early diastole (LASct), reservoir strain in systole (LASr), and contraction strain in late diastole (LAScd)—is known as the LA strain. The first “0” reference point was the LV end-diastole. These strains are LASr, which is defined as the difference between the end-diastole and filling onset; LAScd, which is defined as the difference between the filling onset and the atrial contraction onset (prior to the Doppler A-wave onset); and LASct, which is defined as the difference between the filling onset and the atrial diastole end. The average of the relevant values from both views was used to estimate the global strain (Figure 2) [13].

Strain echocardiographic imaging, in general, is limited by inter-vendor variability and operator expertise, which can affect measurement accuracy and repeatability. The technique’s efficacy is also affected by frame rate settings and extrinsic mechanical variables like heart rate and patient mobility [14,15]. To enhance the reliability of longitudinal strain (LA strain) measurements, we employed a robust approach involving three independent reviewers alongside interobserver and intraobserver methods. By utilizing multiple reviewers, we minimized individual biases and ensured a comprehensive analysis of the data. Interobserver methods allowed for the assessment of variability among different reviewers, while intraobserver methods evaluated consistency within the same reviewer over time. This multifaceted strategy not only bolstered the accuracy of our LA strain measurements but also instilled greater confidence in the findings, ultimately contributing to more reliable clinical assessments.

## 5. Statistical Analysis

The Shapiro–Wilk test and histograms were used to evaluate the normality of the data distribution, and SPSS v27 (IBM©, Armonk, NY, USA) was utilized for the statistical analysis. Using a one-way ANOVA with post hoc analysis for the five groups and an unpaired Student’s *t*-test for the two groups, the quantitative parametric data were evaluated. They are shown as mean and standard deviation (SD). The AUC for the diagnostic efficacy of E/A, LAVI, LA reservoir strain, and LA strain conduit in significantly detecting LV diastolic function in patients with CKD was estimated using receiver operating characteristic (ROC) curve analysis. The frequency and percentage of the qualitative variables were examined using the Fisher’s exact test or the Chi-square test, as applicable.

## 6. Results

The study participants was categorized into five groups on the basis of GFR i.e., GR1 = 20 (%), Gr2 = 24 (%), Gr3 = 72 (%), Gr4 = 64 (%), and Gr5 = 40 (%). The mean age of study contributors was 62.26 ± 9.8 years. Out of 220 participants, 112 (51%) were male, and 108 (49%) were female patients. The distribution of study participants according to the GFR groups is presented in Table 1. The patients’ maximum mean age was observed in Gr5 group, i.e., 75.6 ± 8.7 years, and the minimum was observed in the Gr2 group, i.e., 51.4 ± 14.8. In total, 70.8% and 75% male patients belonged to the Gr2 and Gr1 groups, respectively. In total, 62.5% female patients were in Gr4 group. Hypertension was indicated in 67.5% of cases in the Gr5 group. In total, 85% of patients in the Gr1 group had diabetes, and 50% patients in the Gr1 and Gr2 groups had a smoking history. In addition, 50% of patients in the Gr5 reported dyslipidemia. Diastolic dysfunction grades 2 and 3 were seen in the Gr4 (59.4% and 20.3%, respectively) and Gr5 groups (37.5% and 57.5%, respectively). None of the patients in the Gr1 and Gr2 groups reported grade 2 or 3 diastolic dysfunction. The means and standard deviations of the studied markers, i.e., E/A, average E/e′, TR velocity, and LAVI, are depicted in Figure 2. The maximum mean values among all markers were observed in the Gr5 group. LA reservoir strain was significantly different among the studied groups (*p* < 0.001). It was significantly elevated in Gr1 compared with Gr3, 4, and 5 (*p* value < 0.05) and insignificantly different compared with Gr2. It was significantly higher in Gr2 than Gr3 and 5 (*p* < 0.05), insignificantly different in Gr1 and 4, significantly lower in Gr3 compared with Gr 1, 2, and 4 (*p* < 0.05), significantly less in Gr4 than Gr1, significantly greater in Gr4 than Gr3 (*p* < 0.05), and s significantly lower in Gr5 than Gr1 and 2 (*p* < 0.05). LA strain conduit was significantly different among the studied groups (*p* < 0.001). It was significantly lower in Gr1 than Gr2, 3, 4, and 5 (*p* < 0.05). Gr2 had significantly lower values than Gr 3, 4 and 5 (*p* < 0.05). It was significantly less in Gr3 than Gr4 and 5 (*p* value < 0.05) and was significantly reduced in Gr4 than Gr5 (*p* < 0.05). LA strain contraction was insignificantly different among the studied groups, as shown in Table 1.

**Figure 3:**
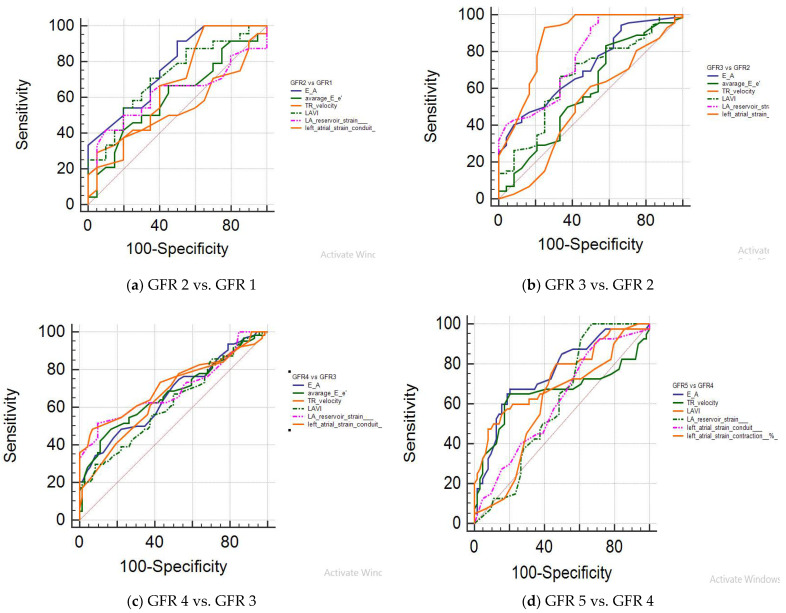
ROC curves of the studied markers in the studied groups.

The diagnostic performance of the studied markers among the GFR groups is described in Table 2. The GFR groups were subgrouped and compared based on the studied markers. The sensitivity, specificity, and AUC are indicated. The findings of the study showed statistically significant findings for E/A, LAVI, and LAScd among all subgroups (*p* ≤ 0.01). The comparison of GFR 3 vs. GFR 2 showed statistically significant findings for LASr (*p* ≤ 0.01). The comparisons of subgroup GFR 4 vs. GFR 3 and GFR 4 vs. GFR 5 showed >90% specificity for LAVI, LASr, and LAScd, and statistically significant findings for both subgroups were observed with all of the markers (*p* ≤ 0.01). An AUC more than 0.7 was seen for E/A and LAVI in GFR 2 vs. GFR 1; E/A, LASr, and LAScd for GFR 3 vs. GFR 2; LAScd for GFR 4 vs. GFR 3; and E/A and LAVI for GFR 5 vs. GFR 4. The receiver operating (ROC) curves of GFR subgroups using the studied markers are shown in Figure 3. The AUCs of the subgroups using the remaining markers were <0.7, as shown in Table 2 and Figure 3.

**Figure 4:**
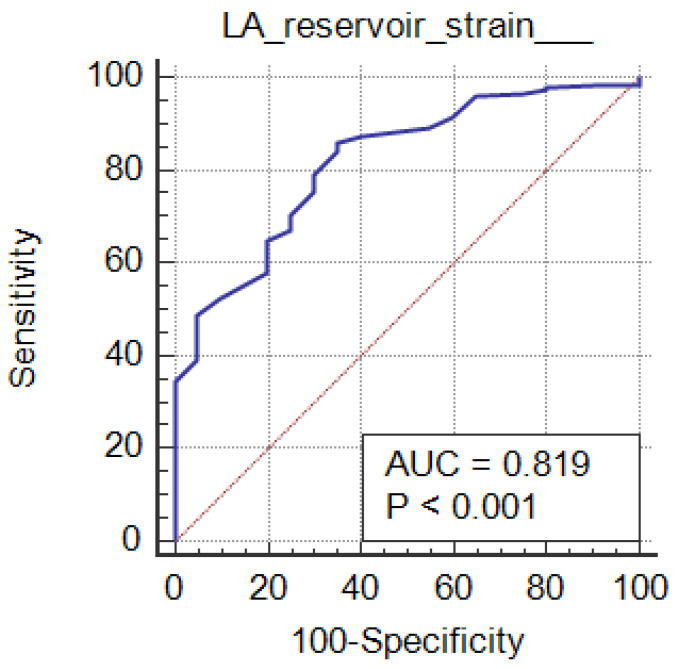
ROC curve of the LA reservoir strain in the studied groups.

**Figure 5:**
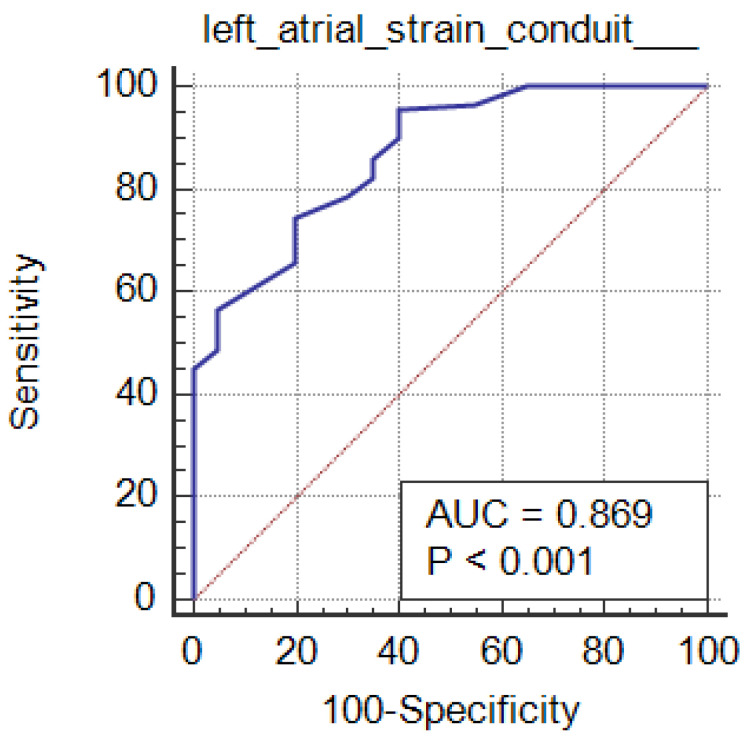
ROC curve of the LA conduit strain in the studied groups.

**Figure 6:**
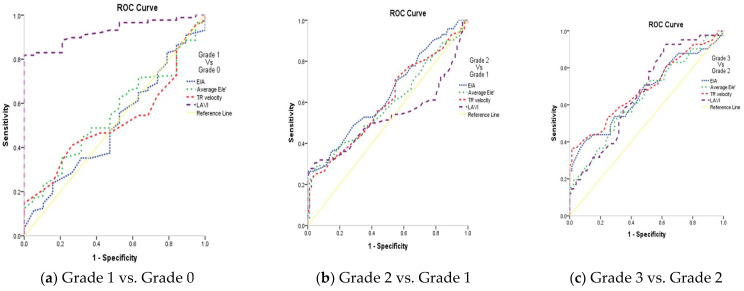
ROC curves of the studied markers based on grades of the diastolic function.

The diagnostic performance of the studied markers among the grades of the diastolic function is mentioned in Table 3. Based on comparisons of grades with studied markers, their sensitivity, specificity and AUC revealed statistically significant findings for grade 3 vs. grade 2 (*p* ≤ 0.01). All the markers except LAVI showed significance for grade 2 vs. grade 1 (*p* < 0.05). Grade 1 vs. grade 0 showed statistical significance with LAVI (*p* < 0.0001) only. LA reservoir strain and LA strain conduit can significantly diagnose LV diastolic function in CKD patients (*p* < 0.001; AUC = 0.819 and 0.869, respectively) at cutoffs of ≤36 and ≥23, with 86% and 95.5% sensitivity, 65% and 60% specificity, 96.1% and 96% PPV, and 31.7% and 57.1% NPV, respectively. Table 3, Figure 4; Figure 5. LA strain contraction cannot diagnose LV diastolic function in patients with CKD, as shown in Table 3, 

ROC curves of studied markers among grades of diastolic function are depicted in Figure 4. An AUC = 0.926 was observed with LAVI among grade 1 vs. grade 0 (0.9 is considered excellent in diagnosing patients with and without the disease) as shown in Figure 6a. The LAVI curve (purple dotted line) is above the diagonal line. Other markers had AUCs of 0.5–0.6 among the grades of the diastolic function, which suggests no discrimination in diagnosing the disease, as shown in Figure 6.

## 7. Discussion

LA strain is very reproducible and feasible, and it has lately become a strong measure for assessing LVDD [16]. We aimed to address the role of LA strain value versus tissue Doppler Echo and the LA volume index in the evaluation of LV diastolic function in CKD patients.

In this study, LA strain was more sensitive and accurate than LAVI, especially in early kidney disease stages, as early diagnosis of the disease gives better prognosis. In addition, we reported that conduit strain and LA reservoir strain had stronger associations with early stages of kidney disease than LA volume index. LA reservoir and LA strain conduit can significantly diagnose LV diastolic function in CKD cases (*p* < 0.001 and AUC = 0.819 and 0.869 respectively) at cutoffs of ≤36 and ≥23, with 86% and 95.5% sensitivity, 65% and 60% specificity, 96.1% and 96% PPV, and 31.7% and 57.1% NPV, respectively. Conduit strain and LA reservoir have been detected in few ESRD studies with adjudicated outcomes.

In unadjusted analyses, both overall mortality and cardiovascular mortality are associated with left atrial strain, according to Tsai et al. [17]. A study by Papadopoulos et al. that included 79 individuals with end-stage renal illness has shown that the left atrial reservoir strain was a predictor of paroxysmal atrial fibrillation [18]. Gan et al. did a novel study on pre-dialysis CKD stages 3 and 4. They discovered that the LA reservoir strain predicts CKD stages and overall mortality with a high degree of accuracy (AUC 0.84). After making adjustments for comorbidities and echocardiographic indicators, the connection between LA reservoir strain and the composite result was found to be substantial [19]. The average LA reservoir strain (24  ±  7%) was much lower than the typical LA reservoir strain (>39%) found in a meta-analysis of healthy populations, according to Pathan et al. [20]. However, it was similar to heart failure cases with HFpEF in the Treatment of Preserved Cardiac Function HF with an Aldosterone Antagonist (TOPCAT) study [21] and similar to CKD patients [22].

In patients with LVEF ≥ 50%, Fan et al. [13]. found that the LA strain was significantly lower in the group of patients who had a high LVEDP. The LA reservoir strain forecasted LVEDP > 16 mmHg with 82.9% specificity and 90% sensitivity at a threshold of 26.7%. This shows that it has a higher diagnostic accuracy than LAScd, E/e′, and LASct.

During the initial LVDD phase, the passive early transmitral diastolic flow is reduced as a result of diminished ventricular compliance and elevated LVEDP. Subsequently, the atrial pump function is improved to balance for the LV filling. The compliance of the LA could be compromised as a result of the elevated atrial pressure, which is necessary to maintain cardiac output as LV distensibility continues to decrease. As a result of persistently increased LVEDP and lowered LA compliance, LA function is impaired [23]. This might be the fundamental mechanism that explains the negative relationship between left atrial strain and left ventricular end-diastolic pressure.

Because of the close relationship between the two chambers, LV pathological abnormalities might lead to LA function degradation. In patients with CKD, the LA is a chamber that is more likely to accumulate fluid and have increased LV filling pressure [5].

Left atrial cardiomyopathy, frequently resulting from diastolic dysfunction, is marked by structural and functional changes in LA, potentially resulting in various cardiovascular problems [24]. Diastolic dysfunction impairs the heart’s capacity to relax and adequately fill, raising pressure in the left atrium and facilitating its remodeling. Several studies indicate a substantial association between compromised left atrial mechanics and the existence of left atrial fibrosis [24,25,26,27]. This fibrosis intensifies dysfunction, establishing a vicious circle that may impair cardiac output and elevate the risk of AF and other arrhythmias. Comprehending these interactions is essential for formulating tailored options that minimize the effects of diastolic dysfunction on left atrial health [26].

Angiotensin-converting enzyme inhibitors (ACEIs) and angiotensin II receptor blockers (ARBs), in particular, are antifibrotic medications that have shown promise in promoting left atrial reversal remodeling, especially in patients with diastolic dysfunction and concomitant fibrosis [27]. These drugs can lower left atrial pressure, alleviate fibrosis, and enhance overall left atrial function. In individuals with CKD, the administration of ACE inhibitors or angiotensin receptor blockers is warranted to control hypertension, diminish proteinuria, and impede the advancement of kidney disease [28]. Furthermore, these medications may be advantageous for CKD patients with concomitant heart failure or left atrial enlargement because they can promote cardiac remodeling and reduce the risk of arrhythmias, hence enhancing both cardiac and renal outcomes. Cautious monitoring is crucial to address any deterioration of renal function and electrolyte disturbances in this group [27,29]. Recently, gliflozins, SGLT2 inhibitors, are promising treatments for type 2 diabetes mellitus, especially in cardiovascular health. These drugs reduce left ventricular hypertrophy and improve diastolic function while improving glycemic management [30]. Managing diabetes and chronic renal disease with gliflozins may improve myocardial performance by relieving heart failure symptoms and inducing reverse cardiac remodeling. They reduce diastolic dysfunction, demonstrating strain echocardiography’s ability to detect heart abnormalities early in this population and improve outcomes [31].

The most prevalent cardiac abnormalities in CKD cases are LV hypertrophy, dilatation, and dysfunction. In previous research, it was discovered that LV hypertrophy was the initial significant cardiac impairment as a result of a consistently elevated plasma urea levels, which becomes increasingly severe as CDK stages progress [32], earlier than dysfunction and dilatation [5]. In our investigation, GLS rose in tandem with the severity of CKD. This phenomena might be explained by uremic cardiomyopathy and the sensitivity of LV strain to preload and fluid overload [33].

The left atrial volume index (LAVi) is a strong predictor of heart failure and cardiovascular events. Moreover, left atrial strain has a novel role in CKD [34]. Blood is extracted from the pulmonary veins and stored in the left atrium during the cardiac cycle. The mitral valve facilitates blood flow from the left atrium to the left ventricle during the diastolic period. The left atrium will use its contractile force to move the remaining blood into the left ventricle [35].

Nguyen et al. [22] did not observe this trend; nevertheless, the LASr and LAScd functions decreased in conjunction with renal function, as seen by the speckle tracking Echo data. These echocardiographic speckle-tracking characteristics may be more susceptible to fluid overload in the early stages of chronic renal disease, as this study indicates that the LA reservoir conduit functions are a significant indicator associated with the severity of CKD. This discovery was consistent with an earlier report that demonstrated that impaired LA and LV strain in CKD patients had no relationship with deteriorating renal function and that eGFR was associated with GLS, LAScd, and LASr [36].

Despite the absence of any differences in conventional Doppler parameters (E/A, E/e′), the diastolic myocardial mechanics of the ESRD group were significantly altered in a study that involved 53 ESRD cases and 85 controls. The earSR E/S ratio, apically atrial strain, reverse rotation, and strain rate fraction were significantly reduced in the ESRD group [37]. Additionally, the type of dialysis affects the LA strain parameters according to a single, limited study done by Aksu et al. [38] In contrast to hemodialysis patients, patients who underwent peritoneal dialysis exhibited significantly greater peak systolic strain and LA strain with contraction values.

Before volume changes occur, the LA strain is altered, and it has the potential to predict both systolic and diastolic dysfunction. The association among DD, LA function, and brain natriuretic peptide (BNP) levels before and after maintenance hemodialysis (HD) sessions was evaluated. Thirty HD patients with a sustained ejection fraction were included in the trial. Before HD, BNP readings had a negative correlation with strain, LA volumes, and E/e′.

There has been debate about whether echocardiographic LA abnormalities are more specific in this situation since patients with chronic kidney disease often have at least one comorbidity. Kadappu et al. sought to demonstrate the size and function of the LA by contrasting hypertensive people with normal renal function with CKD cases. Determining the possible additive CKD impact on LA echocardiographic parameters was the goal. Although there were no variations in LAVi between hypertensive and CKD patients, CKD cases had a significant reduction in color tissue Doppler systolic strain, conduit, LA reservoir, and contractile functions. When differentiating the effects of CKD from other possible related comorbidities, LA strain is thus more effective than conventional two-dimensional echocardiographic assessments [39].

Additionally, LA reservoir, LA strain, and contractile function evaluations are highly beneficial in the identification of myocardial involvement in CKD, as these parameters decrease significantly in CKD cases contrasted to controls. These reductions occur earlier in time than those of LV parameters [40].

Similar to the general population, LA strain seems to have prognostic importance in renal patients. In 243 CKD stage 3–4 patients, the LA reservoir strain was able to independently predict MACCE and cardiovascular death. It was much more accurate than recognized clinical risk ratings (Framingham and atherosclerotic CVD) as well as LV mass, strain, and volume [19].

## 8. Limitations and Recommendations

Our study is limited by several factors, including its single-center nature. To ensure the validity of our findings, it is necessary to confirm their generalizability using large-scale prospective multicenter studies. In addition, the right heart study was not done.

Further investigations, including a greater patient population, encompassing individuals with various CKD stages and accounting for additional clinical and echocardiographic factors, are necessary to validate our findings.

We suggest conducting more trials on larger cohorts of patients with CKD using novel imaging modalities and incorporating other criteria to enhance the precision of LVDD assessment.

## 9. Conclusions

LA conduit strain and reservoir strain are independent markers that are highly valuable in evaluating diastolic function in patients with CKD, representing a superior and more sensitive approach than LAVI and E/e′ for evaluating LVDD, even in the early CKD stages.

## Figures and Tables

**Table 1:** Study participant distribution (descriptive statistics) based on GFR groups.

GFR Groups		Gr1 n = 20	Gr2 n = 24	Gr3 n = 72	Gr4 n = 64	Gr5 n = 40
Age (years)		55.5 ± 10.4	51.4 ± 14.8	60.8 ± 9.3	68.0 ± 5.9 ^aa,bb,cc^	75.6 ± 8.7 ^aa,bb,cc,dd^
Gender	Female (n = 108)	5 (25.0%)	7 (29.2%)	35 (48.6%)	40 (62.5%)	21 (52.5%)
	Male (n = 112)	15 (75.0%)	17 (70.8%)	37 (51.4%) ^aa,bb^	24 (37.5%) ^aa,bb,cc^	19 (47.5%) ^aa,bb,d^
Hypertension		9 (45.0%)	10 (41.7%)	39 (54.2%) ^b^	34 (53.1%) ^b^	27 (67.5%) ^aa,bb,cc,dd^
Diabetes		17 (85.0%)	19 (79.2%)	54 (75.0%) ^a^	36 (56.3%) ^aa, bb, cc^	17 (42.5%) ^aa,bb,cc,d^
Smoking		10 (50.0%)	12 (50.0%)	21 (29.2%) ^aa,bb^	12 (18.8%) ^aa,bb,c^	8 (20.0%) ^aa,bb,c^
Dyslipidemia		1 (5.0%)	2 (8.3%)	6 (8.3%)	18 (28.1%) ^aa,bb,cc^	20 (50.0%) ^aa,bb,cc,dd^
Grade of diastolic function	0	14 (70.0%)	5 (20.8%) ^aa^	0 (0.0%)	0 (0.0%)	0 (0.0%)
	1	6 (30.0%)	19 (79.2%) ^aa^	48 (66.7%) ^aa,b^	13 (20.3%) ^a,bb,cc^	2 (5.0%) ^aa,bb,cc,dd^
	2	0 (0.0%)	0 (0.0%)	19 (26.4%)	38 (59.4%) ^cc^	15 (37.5%) ^c,dd^
	3	0 (0.0%)	0 (0.0%)	5 (6.9%)	13 (20.3%) ^cc^	23 (57.5%) ^cc,dd^
GFR		102.5 ± 7.2	68.4 ± 8.8 ^aa^	39.3 ± 7.4 ^aa,bb^	22.3 ± 4.0 ^aa,bb,cc^	12.5 ± 2.4 ^aa,bb,cc,dd^
E/A		0.6 ± 0.1	0.7 ± 0.1 ^aa^	0.6 ± 0.1 ^bb^	0.8 ± 0.5 ^aa,cc^	1.4 ± 0.7 ^aa,bb,cc,dd^
Average E/e′		10.2 ± 2.2	10.9 ± 2.3	10.3 ± 2.3	12.1 ± 3.1 ^a,cc^	14.7 ± 3.4 ^aa,bb,cc,dd^
TR velocity		2.1 ± 0.5	2.0 ± 0.6	1.9 ± 0.5	2.3 ± 0.8 ^cc^	2.9 ± 1.2 ^aa,bb,cc^
LAVI		29.3 ± 4.8	33.2 ± 4.9	36.1 ± 3.9 ^aa^	38.2 ± 4.4 ^aa,bb,c^	42.6 ± 5.5 ^aa,bb,cc,dd^
LA strain function	LASr (%)	37.1 ± 6.1 ^cde^	33.83 ± 8.95 ^ce^	24.76 ± 5.6 ^abd^	30.17 ± 7.78 ^ac^	27.55 ± 5.67 ^ab^
	LAScd (%)	−23.15 ± 6.63 ^bcde^	−18.96 ± 5.43 ^acde^	−11.04 ± 3.52 ^abd^	−15.08 ± 5.17 ^abc^	−13.15 ± 4.94 ^ab^
	LASct (%)	−15.85 ± 5.31	−15.21 ± 5.23	−16.93 ± 4.84	−15.8 ± 4.42	−14.28 ± 3.96

Age, GFR, E/A, average E/e′, TR velocity, LA strain (lateral, septal, anterior, and inferior), and LAVI are represented as Mean ± SD; the data were analyzed using AVOVA tests followed by Tamhane test as a post hoc test. Gender, HTN, diabetes, smoking, dyslipidemia, and grade of diastolic function are represented as frequency and percent; the data were analyzed using the X2 test. ^a^ *p* value is significantly different compared with GFR group 1. ^b^ *p* value is significantly different compared with GFR group 2. ^c^ *p* value is significantly different compared with GFR group 3. ^d^ *p* value is significantly different compared with GFR group 4. ^e^ *p* value is significantly different compared with GFR group 5. LASr: Left atrial strain reservoir, LAScd: left atrial strain conduit, LASct: left atrial strain contraction.

**Table 2:** Diagnostic performances of the studied markers among GFR groups.

GFR Groups	Studied Markers	Cutoff	Sn. (%)	Sp. (%)	PPV	NPV	AUC	*p* Value
**GFR 2 vs. GFR 1**	**E/A**	>0.56	91.67	50.00	68.7	83.3	0.771	0.0001 **
	**Average E/e′**	>10.2	66.67	55.00	64.0	57.9	0.604	0.2
	**TR velocity**	≤1.5	29.17	95.00	87.5	52.8	0.544	0.6
	**LAVI**	>31.4	70.83	65.00	70.8	65.0	0.718	0.01 *
	**LASr**	≤28	41.67%	90%	83.3%	56.3%	0.625	0.151
	**LAScd**	>−24	70.83%	45%	60.7%	56.3%	0.670	0.044 *
	**LASct**	>−14	50%	75%	70.6%	55.6%	0.522	0.812
**GFR 3 vs. GFR 2**	**E/A**	≤0.55	40.28	91.67	93.5	33.8	0.717	0.0002 **
	**Average E/e′**	≤12.3	83.33	41.67	81.1	45.5	0.585	0.2
	**TR velocity**	≤1.9	61.11	50.00	78.6	30.0	0.509	0.9
	**LAVI**	>35.4	66.67	66.67	85.7	40.0	0.664	0.01 *
	**LASr**	≤35	100%	45.83%	84.7%	100%	0.775	<0.001 **
	**LAScd**	>−16	93.06%	75%	91.8%	78.3%	0.871	<0.001 **
	**LASct**	≤−15	68.06%	54.17%	81.7%	36.1%	0.603	0.138
**GFR 4 vs. GFR 3**	**E/A**	>0.71	48.44	77.78	66.0	62.9	0.667	0.0003 **
	**Average E/e′**	>12.3	48.44	83.33	72.1	64.5	0.673	0.0002 **
	**TR velocity**	>1.7	78.12	47.22	56.8	70.8	0.660	0.0006 **
	**LAVI**	>40.7	29.69	91.67	76.0	59.5	0.623	0.01 *
	**LASr**	>30	51.56%	90.28%	82.5%	67.7%	0.698	<0.001 **
	**LAScd**	≤−16	48.44%	93.06%	86.1%	67%	0.723	<0.001 **
	**LASct**	>−20	78.12%	37.50%	52.6%	65.9%	0.571	0.149
**GFR 5 vs. GFR 4**	**E/A**	>0.8	67.50	79.69	67.5	79.7	0.756	<0.0001 **
	**Average E/e′**	>14.6	65.00	79.69	66.7	78.5	0.698	0.0003 **
	**TR velocity**	>2.7	65.00	79.69	66.7	78.5	0.658	0.01 *
	**LAVI**	>44.5	47.50	92.19	79.2	73.7	0.739	<0.0001 **
	**LASr**	≤18	7.50%	90.62%	33.3%	61.1%	0.607	0.049 *
	**LAScd**	>−7	12.50%	95.31%	62.5%	63.5%	0.611	0.049 *
	**LASct**	>−14	55%	62.50%	47.8%	69%	0.593	0.100

Sn: Sensitivity, Sp: specificity, PPV: positive predictive value, NPV: negative predictive value, AUC: area under curve, and C.I.: 95% confidence interval. * *p* value <0.05 is significant, ** *p* value < 0.01 is highly significant.

**Table 3:** Diagnostic performances of LA strain function and the studied markers among diastolic function grades.

Diastolic Function Grades	Studied Markers	Cutoff	Sn. %	Sp. %	PPV	NPV	AUC	*p* Value
**Grade 1 vs. Grade 0**	**E/A**	>0.66	37.50	52.63	78.6	15.4	0.505	0.9
	**Average E/e′**	>11.4	37.50	68.42	84.6	19.1	0.550	0.4
	**TR velocity**	>2.5	14.77	100.00	100.0	20.2	0.516	0.8
	**LAVI**	>33.7	81.82	100.00	100.0	54.3	0.926	<0.0001 **
**Grade 2 vs. Grade 1**	**E/A**	>0.91	26.39	100.00	100.0	62.4	0.639	0.001 **
	**Average E/e′**	>13.6	29.17	94.32	80.8	61.9	0.591	0.04 *
	**TR velocity**	>2.7	25.00	95.45	81.8	60.9	0.604	0.02 *
	**LAVI**	>42.1	27.78	98.86	95.2	62.6	0.530	0.5
**Grade 3 vs. Grade 2**	**E/A**	>1.55	41.46	88.89	68.0	72.7	0.668	0.002 **
	**Average E/e′**	>13.2	56.10	66.67	48.9	72.7	0.640	0.01 *
	**TR velocity**	>3.6	36.59	98.61	93.8	73.2	0.689	0.0006 **
	**LAVI**	>35.1	92.68	37.50	45.8	90.0	0.667	0.001 **
**LA reservoir strain (%)**		≤36	86%	65%	96.1%	31.7%	0.819	<0.001 *
**Left atrial strain conduit**		>−23	95.5%	60%	96%	57.1%	0.869	<0.001 *
**Left atrial strain contraction**		>−15	43.5%	65%	92.6%	10.3%	0.502	0.983

Sn: Sensitivity, Sp: Specificity, PPV: Positive predictive value, NPV: negative predictive value, AUC Area under curve, C.I.: 95% confidence interval. * *p* value < 0.05 is significant, ** *p* value < 0.01 is highly significant.

## Data Availability

Correspondence and requests for data supporting the study results can be addressed to the primary author.
